# Imputing Parental Genotypes Through Mendelian Imputation: Ethical and Legal Considerations

**DOI:** 10.1007/s10519-025-10250-0

**Published:** 2026-01-08

**Authors:** Margot P. van de Weijer, Emily Bassett, Paul S. Appelbaum, Evan J. Giangrande, Lucas J. Matthews

**Affiliations:** 1 Genetic Epidemiology, Department of Psychiatry, Amsterdam UMC, location University of Amsterdam, Amsterdam, The Netherlands; 2 MRC Biostatistics Unit, University of Cambridge, Cambridge, UK; 3 Department of Psychiatry, Columbia University, New York State Psychiatric Institute, New York, USA; 4 Analytic & Translational Genetics Unit and Psychiatric & Neurodevelopmental Genetics Unit, Massachusetts General Hospital, Stanley Center for Psychiatric Research, Broad Institute of MIT and Harvard, Boston, MA, USA; 5 Department of Medical Humanities & Ethics, Columbia University, New York, USA; 6 The Hastings Center, Garrison, NY, USA

**Keywords:** Mendelian imputation, Ethics, Genotypes, Informed consent, Genetic nurture

## Abstract

Mendelian imputation is a promising method for imputing missing parental genotypes and estimating direct and indirect genetic effects. While the method has clear scientific benefits, so far there has been little reflection on its legal and ethical implications. In this perspective, we discuss these considerations and provide recommendations on transparent use of this method. We argue that imputed genotypes should be considered identifiable, personal data, and that using these data requires informed consent. We distinguish different scenarios where consent may be missing, such as an individual previously not having been contacted for consent, or a person who was contacted but did not reply or did not wish to participate. Informed consent is important across all these scenarios as it allows individuals to decide whether they want to participate in a study. In addition, transparent communication on how people’s data are used is important for public perception of science, and a failure to communicate this may contribute to mistrust. We discuss potential harmful applications of Mendelian imputation, and how robust regulatory frameworks guiding Institutional Review Boards on novel advanced methods such as Mendelian imputation are currently missing. This complicates decision making, especially when researchers argue for public interest as a lawful basis for foregoing consent. We end with a set of recommendations for (1) future genetic research projects for which Mendelian imputation will be possible; (2) research that has already been conducted using this method; and (3) the inclusion of ethical considerations in the publication of novel techniques/analyses.

## Background

Genome-wide association studies (GWASs) have become common practice for identifying single nucleotide polymorphisms (SNPs) associated with human phenotypes, including behaviours and disease. The ‘genome-wide era’ we are currently living has brought many scientific advances. However, it has also raised complex ethical questions and issues around informed consent and the use of genetic data for research beyond the purpose of the original study. One example of this is the use of direct-to-consumer genetic databases to identify individuals, without consumer consent, by US law enforcement agencies (Kennett 2019). In Havasupai Tribe vs. Arizona State University Board of Regents, researchers likewise used the results of blood samples from tribe members in studies on a range of healthcare outcomes beyond those to which tribe members had originally consented (Drabiak-Syed 2010). Thus, while developments of genomic methods have brought us important insights, it is important to reflect critically on these developments and their potential applications to safeguard ethical integrity.

Associations identified through GWAS partly reflect ‘direct’ genetic effects, and we now know that parts of the identified associations reflect ‘indirect’ effects of alleles in parents through environmental pathways (e.g., rearing practices) (genetic nurture). In addition, confounding factors such as population stratification can bias the estimates. It is important to distinguish between these different effects, as a failure to do so can result in an overestimation of genetic effects. When parent-offspring data are available, it becomes possible to distinguish alleles transmitted from parent to offspring from parental alleles not transmitted to offspring. These non-transmitted parental alleles may still be associated with offspring outcomes through their effects on parental phenotypes and thus the rearing environment. While a growing amount of family data is available through national biobanks and population cohorts, data for one or both parents are often missing. One of the solutions that has been proposed to circumvent this issue is the imputation of parental genotypes based on Mendel’s laws of inheritance (Mendelian imputation). This method imputes parental alleles based on the genotypes of relatives by analysing identity-by-descent (IBD, identical alleles/DNA segments that share a common ancestor). Importantly, this differs from genotype imputation where non-genotyped genetic variants are imputed based on linkage disequilibrium within a single individual, which is standard practice in genetics.

While Mendelian imputation has clear scientific benefits, there has not yet been any discussion about its ethical and legal implications. The method requires using data from persons who have provided consent to infer information about an individual who has not provided consent, raising complex ethical questions. That the technique involves genetic data, which are uniquely sensitive and personal, compounds the ethical complexity. While all analyses that include genetic data implicitly contain information on relatives, and while there are other types of analyses (e.g., estimation of within- and between-family effects using sibling data) that inherently tell us something about parental genotypes, Mendelian imputation goes one step further by explicitly modelling and analysing (identifiable) parental genotypes in a way that can be stored and shared. In this paper, we discuss the ethical and legal considerations of using this method, and whether the method could be used for other applications that may potentially be harmful. We conclude with some recommendations on how we can proceed with using this method more transparently and ethically.

## Mendelian Imputation: An Introduction

Imputation of parental genotypes with the purpose of indirect genetic effect estimation was first introduced by Hwang and colleagues (Hwang et al. 2020). Here, its main purpose was to estimate indirect parental genetic effects (effects mediated by the parental phenotype) on offspring (quantitative) phenotypes. Briefly, expected genotypic dosages of parental genotypes are estimated using genotype data from (half)-sibling relative pairs to create ‘virtual’ or ‘best guess’ parental genotypes. For autosomal loci, paternal and maternal genotypes cannot be distinguished meaning that it is assumed that either paternal or maternal genetic effects do not affect the offspring phenotype that is studied. This approach was extended by Tubbs and colleagues (Tubbs et al. 2021) by including nuclear family data (i.e., including parent-offspring data), allowing for imputation of parent-specific missing genotypes. These methods were shown to produce unbiased estimates of maternal, paternal, and offspring genetic effects on offspring phenotypes.

Around the same time, Young and colleagues (Young et al. 2022) published a similar method where two types of imputation are described: (1) imputation from sibling pairs, and (2) imputation from parent-offspring pairs. The method is similar to the Tubbs et al. (2021), but additionally includes information on neighbouring SNPs to increase imputation accuracy. Here, we briefly describe these approaches but advise reading the original paper for a detailed explanation. Where imputation is from sibling pairs, the sum of maternal and paternal genotypes is imputed as we cannot determine the parent-of-origin from sibling data only. How many parental alleles can be observed depends on how many alleles are shared via IBD by the siblings. This differs from identity-by-state (IBS), which refers to identical alleles/DNA segments that do not necessarily share a common ancestor. Therefore, while two alleles that are IBD must always also be IBS, the reverse is not always true. The process of determining which alleles are inherited together from a common ancestor is called phasing. Given knowledge of the IBD-state in two siblings, we can infer the following about the parental alleles:

- When zero alleles at a given locus are IBD (IBD0), the siblings have inherited two different alleles from their parents. In this case, all four parental alleles are observable. To illustrate: in an IBD0 sibling pair, at a given locus, one sibling has inherited alleles A and A, whereas the other has inherited alleles a and a. The parental genotypes at this locus must be: Aa and Aa.
- When 1 allele is IBD (IBD1), three parental alleles are observable: the one shared by the siblings and two unshared alleles. In this scenario, when both siblings are heterozygous, such as the case in the example above, which allele is shared IBD is underdetermined - it could be either A or a. Information from neighbouring phased SNPs can be used to identify the haplotype (i.e., set of alleles that were inherited together from a single parent) shared by the siblings and, in turn, which allele at the target SNP is shared.
- When two alleles are shared IBD (IBD2), one may observe only two parental alleles. Since one cannot infer the unobserved parental alleles, they are imputed with the (population) frequency (f) of the alleles at that locus.

As the number of siblings increases, the recovery of the combined parental genotype improves until it reaches full recovery.

For imputation from parent-offspring pairs, the genotype of one parent can be imputed based on a proband and the other parent. In the case of imputation of the paternal genotype, one half of the genotype is known and the other half is imputed with expectation f. When both mother and proband are heterozygous, phasing data can be used to infer which allele is the paternal allele. When data on two or more siblings are available, the recovery of the missing parental genotype improves. The extent to which these virtual genotypes correlate to actual parental genotype depends on factors such as the number of included siblings and IBD state variability (i.e., the extent to which alleles are shared IBD0, 1 or 2 across the different loci). These imputed genotypes have been shown to produce unbiased estimates of parental genotypic effects, and of direct genetic effects (Hwang et al. 2020; Young et al. 2022).

Imputed parental genotypes are used in the context of direct and indirect genetic effect estimation (Baker et al. 2024; Kweon et al. 2025; Wu et al. 2021). The imputed genotypes can be used for family-based GWAS, where summary statistics are computed for direct effects, indirect non-transmitted effects, and population effects. In this case, a joint regression is performed onto the proband’s genotype, the father’s imputed or observed genotype, and the mother’s imputed or observed genotype. In addition, the imputed genotypes can be used for polygenic score analysis based on observed or imputed genotypes, and for performing family-based polygenic score analysis. Here, the regression coefficient of the proband’s polygenic score is estimated while controlling for the parental polygenic scores as an estimate of the direct genetic effect and additionally estimate the maternal non-transmitted and paternal non-transmitted coefficients.

Importantly, the imputed parental genotypes are currently used as part of analyses that treat the family as the fundamental unit of analysis, rather than the individual. Polygenic scores are calculated for the imputed parent, but are used to separate direct from indirect genetic effects on offspring outcomes.

## Legal and Ethical Considerations

### Informed Consent for Genetic Data

Informed Consent is both a legal and ethical requirement for research involving human participants. For consent to be informed, a participant needs to understand the purpose and procedures of a study. Collection of any type of identifiable or personal data requires informed consent. Personal or identifiable, in this context, refers to information relating to an identified or identifiable living individual. Personal data can be de-identified or pseudonymised, but so long as the subject of the data can be re-identified, they are still classified as personal data. Only when data are irreversibly anonymised in such a way that they cannot be linked back to an individual are the data no longer considered personal. Insofar as it refers to the genotype of a single individual, genetic data cannot be anonymised in such a way. Yet, there are cases where genetic information is not identifiable, such as when it is aggregated (e.g., summary statistics - but even then there remains some degree of residual identifying information, as summarised in Craig et al 2011), or when the data contain only a subset of genetic markers and are anonymised.

Genetic data are often assigned a “special category” status because these data have the potential to create significant risks to an individual’s rights and freedoms. Under the General Data Protection Regulation (GDPR), the European Union’s data privacy and security law, special category data are “Personal data revealing racial or ethnic origin, political opinions, religious or philosophical beliefs, trade union membership; and the processing of genetic data or biometric data for the purpose of uniquely identifying a person; data concerning health or data concerning sex life or sexual orientation”. In the case of genetic data, data processing is linked to a potential risk of racial or ethnic discrimination, discrimination on the basis of health status (Chapman et al. 2020), and a threat to one’s right to bodily autonomy. Special status data are thus perceived as more sensitive than other types of data and should be treated with greater care.

### Informed Consent for Imputed Genotypes: Legal Considerations

From a legal perspective, it is important to assess whether imputed genetic data fall under the category of personal data or even ‘special category’ personal data. Concerning the latter, the first question to answer is whether imputed genotypes should be considered identifiable data. To answer this question for imputed genotypes, we need to distinguish between imputation when a single parental genotype is missing and when both parental genotypes are missing. When both parents are missing, what is imputed is an average of both parents. This is a likely scenario for non-family-based cohorts and biobanks such as the UK Biobank. Given that this is a sum that does not correspond to an actual person, it can be said that the data are not identifiable. In addition, it seems that the data do not qualify as personal, as the combined genotype does not relate to an identifiable living individual.

When only one parent is missing, however, answering this question is more complex. It cannot be argued that these data are anonymised when they are linked to a proband, and it can easily be inferred to whom this imputed genotype is being attributed. Even if imputation is not entirely accurate, the data are still linked to a particular person and can have negative effects, such as a risk of discrimination, on that person. This still holds if the data are (administratively) linked to the genotyped individual (i.e., proband) and not to the imputed parent, as it is clear to whom the imputed data are assigned. In this case, we would expect it to be necessary that the imputed individual provides informed consent, as it is identifiable, personal data.

Two arguments may be made in favour of consent by only the genotyped proband being sufficient. First, the only certainty in the imputed parental genotype is the half that was transmitted from the missing parent to the genotyped offspring, and this half was obtained using informed consent (from the genotyped individual). Second, we may classify the imputed genotypes as a form of “family history” data, but without an opt-out to not supply these data. In both scenarios, it is questionable whether the informed consent provided by most people who contribute data to biobanks and other repositories covers this specific application of the data. What is extracted is part of the data transmitted by a specific, unconsented individual, an application unlikely to be reasonably covered in informed consent procedures: most individuals who contribute genetic samples will not be aware that their data can even be used in this way. This issue does not only pertain to parent-specific imputation, but also for imputation of the combined parental genotype.

If we assume imputed genotypes are personal, identifiable data, are there circumstances in which the data can be used without explicit consent? Under Article 6(1)(e) of the GDPR, a lawful basis for the processing of personal data (without explicit consent) is ‘public interest’, where: “processing is necessary for the performance of a task carried out in the public interest or in the exercise of official authority vested in the controller”. In a research context, this means there must be an urgent social need for processing personal data, leading to an explicit increase in knowledge in the interest of society. Public interest is also specifically mentioned concerning the processing of special categories of personal data (such as genomic data, Chap. 2, article 9), where consent is not required if (g) “processing [of special category data] is necessary for reasons of substantial public interest, on the basis of Union or Member State law which shall be proportionate to the aim pursued, respect the essence of the right to data protection and provide for suitable and specific measures to safeguard the fundamental rights and the interests of the data subject”. Similarly, in the United States, the most relevant provision of the ‘Common Rule’ regulating most federally funded medical research states that Institutional Review Boards (IRBs) may permit a waiver of consent under the conditions that: “(i) The research involves no more than minimal risk to the subjects; (ii) The research could not practicably be carried out without the requested waiver or alteration; (iii) If the research involves using identifiable private information or identifiable biospecimens, the research could not practicably be carried out without using such information or biospecimens in an identifiable format; (iv) The waiver or alteration will not adversely affect the rights and welfare of the subjects; and (v) Whenever appropriate, the subjects or legally authorized representatives will be provided with additional pertinent information after participation”(HHS regulations for the protection of human subjects 45 CFR 46.101(f)(3)). Whether a specific research use of the data qualifies as public interest and whether the fundamental rights of the data subjects are sufficiently safeguarded (under the GDPR) or whether the multi-part requirements of minimal risk, necessity, and protection of rights are met (under the Common Rule) will vary and need to be assessed on a case-by-case basis. In the specific research context of this perspective, whether the use of imputed genotypes for dissecting direct from indirect genetic effects has a lawful basis for processing can be debated and depends on the context and specific research topic. In addition, we provide examples for the EU and US, but similar exemptions may not be made in other continents and countries.

### A Note on Accuracy

It may be argued that since the ‘true’ parental genotype is not genotyped, but a less accurate version of it, the legal and ethical standards we hold for genotype data may not directly extend to imputed genotypes. As mentioned above, the accuracy of this type of imputation varies depending on the number of siblings included and IBD state variability. However, it is fair to assume that a substantial part of the imputed genotype reflects the true parental genotype, especially when multiple siblings’ genotype data are included. In addition, regardless of how accurate the imputation is, if we consider (single-parent) imputed genotypes identifiable data, we should uphold the same legal and ethical standards we do for other types of identifiable data. Therefore, we emphasise that the potential inaccuracy of imputed genotypes should not serve as a justification for a less stringent ethical evaluation.

### Degrees of Non-Consent and Family-Based Biobanks: Ethical Concerns

In a recent perspective by Davies and colleagues (Davies et al. 2024), the importance of family-based sampling for biobanks is outlined. The authors explain how the inclusion of families in biobanks has the added benefit of reducing or eliminating confounding due to familial and demographic factors. With respect to recruitment, the authors note that “Tracking such family members can be time-intensive, costly and sometimes impossible. Nevertheless, available family members can sometimes provide phenotypic information on missing individuals, and missing genotypes can be imputed using relatives in a way that provides unbiased estimates of direct genetic effects”. While we certainly agree that the inclusion of family members in biobanks is important for scientific purposes, it is important to stress that genotype imputation cannot be implemented as a data collection replacement merely because recruitment would be time-intensive or costly.

There are five scenarios where consent may be missing (ignoring potential missing consent due to administrative or technical errors): (1) the individual cannot be contacted for consent because they are deceased, (2) the individual was not contacted for consent, (3) the individual was contacted for consent, but did not reply (no response), (4) the individual was contacted for consent and replied that they did not wish to participate, and (5) the individual originally consented but withdrew consent. As an example, in the UK Biobank, only approximately 500,000 participants were recruited out of 9.2 million invited individuals (a rate of 5.47%). It is unclear to what extent this reflects the response rate (scenario 3) versus the participation rate (scenario 4) (Allen et al 2012). In 2024, the UK Biobank reported a total of 1,681 withdrawn consents (0.2% - scenario 5).

From a legal perspective, consent for research only applies to living individuals and thus not to individuals imputed under scenario one (i.e., post-mortem). Ethically, while there is no risk of direct negative effects for deceased individuals, it is still important to respect individuals’ wishes, even after they have passed. If an individual had expressed an unwillingness to participate in genetic research while they were still alive, this decision can be used to inform participation in research after they have died. However, if such information is not available, we can treat the missing consent of that person similar to scenario 2 or 3.

In scenario 2, there can be several reasons why a person was not contacted for participation, while their relatives were. For example, they may not be part of the same study, cohort, or biobank as their family members. This could be because the study/cohort focuses on a specific group of people (e.g., only women, individuals with a certain diagnosis, people born in a certain period). However, in scenario 3, they were contacted to be part of the study, cohort, or biobank, but did not respond. While non-response occurs for different reasons, their non-response may indicate that they did not wish to participate. In the most problematic cases (scenarios 4 and 5), individuals were contacted for participation, but the reason for their missing data is that they explicitly indicated they did not want to participate (anymore). This is certainly not an unthinkable scenario for family-based cohorts where entire families are invited for genotyping, but not all family members respond or consent. Setting aside potential legal issues with imputing genotypes of individuals who have explicitly declined consent, there is an ethical issue here: the imputation goes directly against the wishes of the respective individual, who becomes an unwilling research participant. Similarly, if individuals have withdrawn consent, they have explicitly indicated they no longer wish to contribute to this type of research. To our knowledge, current studies using Mendelian imputation do not consider the reason behind missing data before imputation. Regardless of the reason behind missing consent and genotype data, informed consent is a crucial aspect of allowing individuals to incorporate their own values and beliefs in making autonomous decisions to (not) participate in research. Currently, imputation in scenarios 2–4 fails to adhere to these crucial ethical standards.

### Trust in Scientific Research(ers)

Are there other considerations, in addition to the legal and ethical concerns, that should be taken into account with respect to the use of Mendelian imputation? In the next section, we discuss whether there is a potential for (harmful) application beyond its current application for direct and indirect effect estimation. Here, we address another consequence that should be considered: the consequences for trust in scientific research(ers). A recent comprehensive review on trust in scientists and their role in society across 68 countries found more optimistic results for trust than expected: in general, trust in scientists was moderately high (Cologna et al. 2025). With respect to scientific integrity, 57% of people believed that most scientists are honest, 11% believed that they are dishonest, and 31% selected the 1–5 scale at midpoint. In addition, only 42% of respondents believed that scientists pay attention to others’ views somewhat or very much. As a recommendation, the authors write the following: “Public perception of scientific integrity [.] is somewhat high, but perceptions of scientists’ openness are lower. Therefore, scientists wishing to gain public trust could work on being more receptive to feedback and more transparent about their funding and data sources, and invest more effort into communicating about science with the public—which we found to be desired by 83% of respondents”. We believe that disclosure about how an individual’s data can be used is another important aspect of this openness and that a failure to do so will be detrimental to trust and public perception of scientific integrity. While individuals are mostly aware that they share DNA with their relatives and that by sharing their DNA for research purposes they are also sharing part of their relatives’ DNA, we cannot reasonably assume that they are also aware of methods such as Mendelian imputation that allow for (partial) reconstruction of the DNA of their relatives. Although papers describing these methods are publicly available, they are too technical for a general audience. A failure to explain these methods and the possibility that their data will be used for these purposes (and why this benefits science) may contribute to mistrust.

### Ethical Concerns Over Potential Harmful Applications

Currently, Mendelian imputation has only been used to boost power when estimating direct and indirect genetic effects. In addition to the ethical considerations that we have discussed in that context, it is also important to consider whether (1) Mendelian imputation for direct genetic effect estimation can be used in sensitive research areas that require additional caution and (2) whether the method could be extended to other types of analyses that raise novel ethical questions.

With respect to the first point, Mendelian imputation for better estimating direct genetic effects could be applied in sensitive, highly controversial areas of research that themselves have extensive ethical, legal, and social implications. For example, Young and colleagues speculate that Mendelian imputation could be useful in polygenic embryo selection: “ [.] for prediction of differences between embryos, only direct effects are relevant, so selecting embryos using population effects would perform poorly compared to using direct effects (assuming equal precision of direct and population effect estimates) and could introduce confounding related biases. Confounding related biases can also lead to spurious inferences in Mendelian randomization and studies of selection. If NTCs [ed.: non transmitted coefficients] are substantial and imperfectly correlated with direct genetic effects, then prediction accuracy could be increased by including predictors based on NTCs of parental genotypes in addition to predictors based on proband genotypes”. Polygenic embryo selection is a highly controversial topic of scientific interest and discussion (Furrer et al. 2024; Polyakov et al. 2022) that itself presents challenging ethical questions and carries a significant risk of harm. Given that imputed genotypes may facilitate the execution of sensitive procedures such as (polygenic) embryo selection, deliberate consideration of the ethical considerations detailed in this perspective is warranted. Relatedly, it could be the case that the parental phenotype can be assumed based on the child’s phenotype or genotype. Think, for example, about Mendelian disorders, i.e., disorders that are caused by mutations in a single gene. Autosomal dominant disorders such as Huntington’s disease could be unveiled through genetic analyses, but may not yet have manifested in both parent and offspring. How biobanks and cohort studies deal with incidental genetic findings is usually covered in participant information leaflets and informed consent, but these do not cover imputed individuals.

Regarding the second point, Mendelian imputation may enable future methodological extensions beyond simply improving direct genetic effect estimation. In research scenarios where (imputed) parents are the fundamental unit of analysis, imputed parental genotypes may be useful. Consider a study in which probands are asked about family history (e.g., UK Biobank asks participants: ‘Has/did your father (or mother) ever suffer from…?’ followed by a list of illnesses), or a register-based study in which parental phenotypic data are available. In either scenario, Mendelian imputation could be used to impute parental genotypes, which could then be combined with the indirectly ascertained phenotypes to enable genetically informed analyses of the parents, such as GWAS or individual-level Mendelian randomization. Although Mendelian imputed genotypes may be of lower quality than directly ascertained genotypes, such an approach is theoretically possible but would clearly violate data protection regulations. Although such approaches should therefore not pass institutional review, there are currently no clear guidelines guiding IRBs; genetic advances often outpace ethical and legislative consideration, let alone regulation.

### Conclusion

In this section, we have discussed several legal and ethical consequences of Mendelian imputation. From a legal perspective, it is important to consider whether imputed genotypes may be viewed as identifiable data for which informed consent is necessary. One way in which these genotypes may not be identifiable is if they are irreversibly anonymised. In the specific case of direct and indirect effect estimation, if these imputed genotypes are not stored in any way, it may be argued that these data are anonymized as re-identification based on the direct and indirect effect sizes only seems highly unlikely. However, even in this scenario, there are several ethical considerations that should be taken into account. For one, it is important to assess the reason behind missing consent, and especially to evaluate whether this person was previously invited to participate or not. In addition, it is important to safeguard people’s autonomy over their own data, which may be violated especially if their imputed data are used in studies in which they do not wish to participate. Other ethical considerations are trust in science and scientific researchers, potential other applications besides direct and indirect effect estimation, and regulations with respect to reporting of serious health outcomes.

## Related Examples

### Family History Reporting

It is interesting to compare the example where individuals are asked to report on their relatives’ disease status to inferring parental genotypes through Mendelian imputation. While individuals are more explicitly providing information on their relatives in these questions than is the case with Mendelian imputation, these situations are also accompanied by potential issues with data privacy and ownership. Indeed, there has been controversy around the ethics of this type of approach. In the beginning of this century, human subject research was temporarily suspended at Virginia Commonwealth University (VCU) in the United States as a direct result of a case where a woman was recruited to participate in a twin study for which she had to fill out a questionnaire that reportedly asked multiple questions related to her father, such as whether he suffered from depression. Concerned about his privacy and lack of informed consent, the father contacted the National Institutes of Health’s Office for Protection from Research Risks (now: the Office for Human Research Protections). After reviewing the case, the office ruled that the IRB did not sufficiently consider whether family members were human subjects in this research. Eventually, human subject research was temporarily suspended at VCU after reviewing the IRB procedures, stating that the IRB did not adequately document its monitoring of research protocols (Botkin 2001).

The critical issue in this case was that it is important that IRBs consider who is a human subject in the research/ study under consideration, and once all subjects have been identified, whether each subject’s rights are protected and/or whether there is minimal risk for these subjects. This relates to the issues described in the sections above, of whether and when imputed data count as personal data, and if so, under what circumstances these data may be used without informed consent. Currently, Mendelian imputation studies also need to pass local IRBs, which decide if all conditions have been met. However, methods such as these are so novel that no robust regulatory frameworks are available to guide institutional review boards when making these decisions. This may be especially difficult under the GDPR when public interest is argued as a lawful basis for foregoing consent.

There seems to be a dual-use dilemma for introducing novel methods such as these: on one hand, some researchers believe we should pursue these methods since they could lead to novel discoveries that could aid society. However, it should be noted that Mendelian imputation is merely a means of improving power by rapidly increasing the sample size for existing methods for examining direct and indirect genetic effects, meaning that we could still study these effects with genotyped data from consenting subjects only. In addition, it could also lead to unwanted research practices that violate people’s agency over their data and result in a breach of trust between society and researchers. Therefore, regardless of current application and intentions, careful consideration of potential impacts and the development of strategies to address these in the future is vital for upholding high ethical standards for genomic research.

### iPsych

Throughout the paper, we have only considered situations in which individuals who contributed their samples had provided consent. However, there are also situations in which participants’ genetic data are included without this consent. An example is the Danish iPsych case-cohort sample (Pedersen et al. 2018), which includes individuals from the Danish population born between 1981 and 2008 with a diagnosis of selected mental disorders and a control cohort from the general population. Genotyping is possible through neonatal dried blood spots (‘the heel prick’) from the Danish Neonatal Screenings Biobank. Data are linked using a Central Person Register (CPR) number and researchers who use the data access pseudonymized data, where the link with CPR-number is removed. Ethics approval for a waiver of consent was provided by a regional science ethics committee in 2012. This waiver, which enables analysis of a more representative sample of the population, is acceptable as long as a study does not imply health-related risks or inconvenience participants. However, in 2019, an application to continue extracting DNA was not approved under stricter requirements for waiving informed consent. Nevertheless, the already extracted and sequenced DNA can still be used for research, as it is covered by data protection laws and not biological tissue laws. As a response to critiques of this policy, all individuals included in iPsych very recently (June 20, 2025) received a notification that they are included in the iPsych cohort, with an option to opt out of future research.

Cases such as iPsych are interesting in the context of Mendelian imputation for two reasons. First, it underscores the need to balance individual rights and autonomy with public benefits from valuable scientific research. If we were to follow a similar procedure as taken by iPsych for Mendelian imputation research, we could say that individuals who have already been imputed could opt out of new analyses. A news article published in Science magazine (Inampudi 2025) discussed the controversy surrounding the situation, highlighting the importance of people’s awareness of participation in research and the responses of individuals who feel their privacy has been invaded. For individuals already imputed, this may evoke comparable concerns regarding privacy, indicating that providing these participants with the opportunity to opt out promptly would be preferable.

Second, data from cohorts such as iPsych can potentially be used for Mendelian imputation, adding an extra layer of complexity. Even if these data are anonymous, calculating genetic relatedness in the sample could reveal familial relatedness, which in turn could be leveraged to impute ungenotyped individuals. In this scenario, neither the genotyped nor the imputed individuals provided consent. While researchers may still argue public interest as a reason for pursuing this approach, said interest would have to be exceptionally compelling to override informed consent on multiple levels.

### DeCODE Genetics

An applied example of large-scale imputation of relatives in a population-based cohort is the Icelandic deCODE genetics cohort (Hakonarson et al. 2003). DeCODE genetics is an Icelandic (private) research company that includes around 150,000 genotyped Icelandic individuals, almost half of the country’s population. In addition, deCODE has created an extremely comprehensive genealogy database of the entire country through records dating back as early as the late 1600s. By linking genetic, genealogical, and medical records, deCODE has made huge contributions to genetics research. Nevertheless, the database has also led to controversy and many ethical critiques and debates (Árnason 2016). Relevant to the issue at hand, deCODE combines its measured genotypes with the genealogical database to estimate so-called ‘in-silico’ genotypes of close relatives of genotyped volunteers, who did not consent to participation. While deCODE originally planned to link these imputed genotypes to hospital records, Iceland’s data protection authority (DTA) did not allow for this linkage in the absence of informed consent. As a response, deCODE came up with a novel proposal where measured genotypes and genealogy data would be used to generate imputed genotypes (supplemented by medical records) in genetic analyses without actually storing these imputed data, which was permitted by the DTA. However, even under this model, the approach is still controversial, especially given the potential for reidentification, the fact that individuals may not wish to participate in specific research studies due to other concerns than re-identification (e.g., religious reasons), and a lack of attempts to obtain informed consent, especially in light of privacy and autonomy rights (Gitter 2019).

## Summary and Recommendations

To summarise, several considerations should be taken into account before commencing a project that includes Mendelian imputation. Genetic data are considered identifiable, personal data that are more sensitive and should be treated with even greater care than other data types. It is essential to determine whether imputed parental genotypes should also be considered identifiable personal data, as this would necessitate obtaining informed consent. In addition, it is questionable whether informed consent procedures for the (non-imputed) genotyped individuals cover this use of their data. Another crucial point to consider is the reason behind missing genotype data, with an important distinction between individuals who were not approached for genotyping and individuals who were approached but declined participation (active non-consent). In both cases, there are potential ethical problems, such as whether individuals’ autonomous choice to participate in research is protected. In case of active non-consent, there is additionally a legal question of whether imputation is even allowed. Besides these ethical and legal considerations, a failure to disclose and communicate methods such as these properly could lead to a breach of trust and may have harmful consequences for public perception of scientific integrity. Lastly, potential applications of Mendelian imputation that go beyond analysis on the family unit and downstream consequences in the context of sensitive research topics require strict ethical scrutiny from IRBs. However, robust regulatory frameworks for IRBs when making these decisions in the context of Mendelian imputation are currently missing, and decisions may be especially difficult when public interest is argued as a lawful basis for foregoing consent.

We conclude with a set of recommendations designed to address the ethical and legal considerations discussed.

### Recommendation 1: For future genetic research projects for which Mendelian imputation will be possible, participants who are the targets of imputation should be informed of the possibility and be invited to provide consent before the study takes place. In the absence of a response, an IRB should assess if intermediate consent by relatives and waiver of consent for the imputed person suffice.

With respect to consent and Mendelian imputation, we recommend the following:

1. If possible, assess the reason behind missing consent/ genotype: was the individual previously contacted for genotyping? If the reason behind missing genotyping data is overt refusal (or withdrawn consent), imputation should not occur.
2. Was the individual previously contacted, but did not respond? Assess whether “intermediate consent” by genotyped relatives is sufficient for imputation. Here, the context of the research question should also be taken into account. If the imputed data will be used for contentious topics, the research should be held to greater ethical standards. In addition, the consent process for directly involved participants should include information about this type of application of their data.
3. Has the individual not been contacted before? Before imputation is considered, research participants should first be contacted and asked to consent to imputation (or genotyping).

If public interest is used as an argument for foregoing consent, there should be clear and exhaustive evidence for: (1) the importance of the research question, (2) the steps previously taken to contact individuals for consent, and (3) the necessity for imputation to answer the research question.

It is imaginable that it would not be possible to assess the reason behind missing consent. For example, researchers may not have access to this type of information when using biobank data, and biobanks may not wish to share these data. Since we cannot determine whether the absence of consent indicates non-consent or missing consent, it would be safest to assume non-consent.

### Recommendation 2: Participants whose genotype has been inferred using Mendelian imputation should be informed.

The recommendations provided above pertain to novel research questions. With respect to research already conducted, we recommend that participants whose genotype has been inferred using Mendelian imputation are informed. In case this is practically infeasible (e.g., contact information is unavailable), the genotyped relatives whose data were used for the imputation should be contacted to inform them of this novel application of their data.

### Recommendation 3: Methods papers/tutorials for novel techniques/analyses such as Mendelian imputation should include discussions of ethical considerations.

Novel research techniques and analyses are commonly published, and are often easy to apply, even if researchers are not familiar with the details, due to accessible tutorials that are published along with these methods. What is often missing from these papers and tutorials are ethical considerations to which researchers need to pay attention. We recommend publishing FAQs or supplementary documents that address ethical and legal questions in these scenarios that can help inform not only the researchers, but also research participants and IRBs.

We realize that our recommendations, especially to obtain consent before imputation, are more time- and cost-intensive than the current standard practice. People have a fundamental right to privacy and autonomy, reflected in e.g. the charter of fundamental rights of the European Union, the GDPR, and the Declaration of Helsinki (European Union 2012; World Medical Association 2013). Safeguarding people’s privacy and autonomy is one of the most important responsibilities that researchers carry, and this is important enough to warrant extra time and money. However, another important responsibility of researchers is to perform research that generates the best knowledge possible to benefit the public. It may therefore seem as if these two responsibilities clash, as the best knowledge often comes from large sample sizes, and our recommended approach may require a slower and more expensive effort to reach the largest sample size. It is important to note two things. First, this research can still be conducted, although it will cost more in time and resources. Second, in case of extremely important research, a waiver of consent can be requested in light of the public interest. However, since there are no straightforward criteria for determining public interest, these requests should be scrutinized carefully and not be taken lightly.

New technologies may require new approaches to human subjects protections. We believe that Mendelian imputation is one of those instances.

## Data Availability

No datasets were generated or analysed during the current study.

## References

[R1] Allen N (2012) UK biobank: current status and what it means for epidemiology. Health Policy Technol 1:123–126

[R2] Árnason V (2016) Bioethics in Iceland: recent developments. Camb Q Healthc Ethics 25:421–434 10.1017/S0963180116000074 27348827

[R3] World Medical Association (2013) World medical association declaration of Helsinki: ethical principles for medical research involving human subjects. JAMA 310:2191–2194 10.1001/jama.2013.281053 24141714

[R4] Baker S, Biroli P, van Kippersluis H, von Hinke S (2024) Advantageous early-life environments cushion the genetic risk for ischemic heart disease. Proc Natl Acad Sci USA 121:e2314056121 10.1073/pnas.2314056121 PMC11228495 38917008

[R5] Botkin JR (2001) Protecting the privacy of family members in survey and pedigree research. JAMA 285:207–211 10.1001/jama.285.2.207 11176815

[R6] Chapman CR, Mehta KS, Parent B, Caplan AL (2020) Genetic discrimination: emerging ethical challenges in the context of advancing technology. J Law Biosci 7(1):lz016 10.1093/jlb/lsz016 PMC8249090 34221431

[R7] Cologna V (2025) Trust in scientists and their role in society across 68 countries. Nat Hum Behav 9:713–730 10.1038/s41562-024-02090-5 PMC7617525 39833424

[R8] Craig DW (2011) Assessing and managing risk when sharing aggregate genetic variant data. Nat Rev Genet 12:730–736 10.1038/nrg3067 PMC3349221 21921928

[R9] Davies NM (2024) The importance of family-based sampling for biobanks. Nature 634:795–803 10.1038/s41586-024-07721-5 PMC11623399 39443775

[R10] Drabiak-Syed K (2010) Lessons from Havasupai tribe v. Arizona state university board of regents: recognizing group, cultural, and dignity harms as legitimate risks warranting integration into research practice. J Health Biomed Law 6:175

[R11] Furrer RA (2024) Public attitudes, interests, and concerns regarding polygenic embryo screening. JAMA Netw Open 7:e2410832–e2410832 10.1001/jamanetworkopen.2024.10832 PMC11094562 38743425

[R12] Gitter DM (2019) The ethics of big data in genomics: the instructive Icelandic Saga of the incidentalome. Wash U Glob Stud Law Rev 18:351

[R13] Hakonarson H, Gulcher JR, Stefansson K (2003) DeCODE genetics, inc. Pharmacogenomics 4:209–215 10.1517/phgs.4.2.209.22627 12605555

[R14] Hwang L-D (2020) Estimating indirect parental genetic effects on offspring phenotypes using virtual parental genotypes derived from sibling and half sibling pairs. PLoS Genet 16:e1009154 10.1371/journal.pgen.1009154 PMC7646364 33104719

[R15] Inampudi A (2025) Study used DNA from thousands—without consent. Science 389:552–553 10.1126/science.aeb2395 40773549

[R16] Kennett D (2019) Using genetic genealogy databases in missing persons cases and to develop suspect leads in violent crimes. Forensic Sci Int 301:107–117 10.1016/j.forsciint.2019.05.016 31153988

[R17] Kweon H (2025) Associations between common genetic variants and income provide insights about the socio-economic health gradient. Nat Hum Behav 9:794–805 10.1038/s41562-024-02080-7 PMC12018258 39875632

[R18] Pedersen CB (2018) The iPSYCH2012 case–cohort sample: new directions for unravelling genetic and environmental architectures of severe mental disorders. Mol Psychiatry 23:6–14 10.1038/mp.2017.196 PMC5754466 28924187

[R19] Polyakov A (2022) Polygenic risk score for embryo selection— not ready for prime time. Hum Reprod 37:2229–2236 10.1093/humrep/deac159 PMC9527452 35852518

[R20] Tubbs JD, Hwang L-D, Luong J, Evans DM, Sham PC (2021) Modeling parent-specific genetic nurture in families with missing parental genotypes: application to birthweight and BMI. Behav Genet 51:289–300 10.1007/s10519-020-10040-w 33454873

[R21] European Union (2012) Vol. /C 326/02 391–407 (Official Journal of the European Union, 2012)

[R22] Wu Y (2021) Estimating genetic nurture with summary statistics of multigenerational genome-wide association studies. Proc Natl Acad Sci USA 118:e2023184118 10.1073/pnas.2023184118 PMC8237646 34131076

[R23] Young AI (2022) Mendelian imputation of parental genotypes improves estimates of direct genetic effects. Nat Genet 54:897–905 10.1038/s41588-022-01085-0 PMC9197765 35681053

